# NMR metabolomics reveals metabolic alterations in a novel mouse model of neurodegeneration

**DOI:** 10.3389/fnins.2026.1776973

**Published:** 2026-02-12

**Authors:** Grace V. Mercer, Haley Adams, Drew P. Locke, Sophie Kiefte, Nikita Harvey, Rachel Coffey, Céline M. Schneider, Lindsay S. Cahill

**Affiliations:** 1 Department of Chemistry, Memorial University of Newfoundland, St. John’s, NL, Canada; 2 Discipline of Radiology, Memorial University of Newfoundland, St. John’s, NL, Canada

**Keywords:** metabolomics, mouse, MRPL3, neurodegeneration, nuclear magnetic resonance, sex differences

## Abstract

**Introduction:**

The relationship between brain metabolism and neurodegenerative diseases is poorly understood. To investigate the pathophysiology of neurodegeneration, we used decrepit (*dcr*) mice, a mouse model with a mutation in a mitochondrial associated gene (mitochondrial ribosomal protein L3, *Mrpl3*) that results in reproducible, adult-onset degeneration of the brain.

**Methods:**

Metabolite profiles were determined using ^1^H magic angle spinning nuclear magnetic resonance (600 MHz spectrometer) and *ex vivo* tissue samples from five brain regions in female and male *dcr* and healthy control mice (*n* = 39–44 mice/genotype/sex from 44–145 days of age).

**Results:**

The relative concentration of acetate, N-acetylaspartate, gamma-aminobutyric acid, glutamine, and asparagine were decreased in the *dcr* mice compared to controls (*p* < 0.05). When the data was disaggregated by sex, the male *dcr* mice showed decreased relative concentrations of acetate, N-acetylaspartate, gamma-aminobutyric acid, asparagine, and choline-containing compounds compared to controls (*p* < 0.05) while the female *dcr* mice had an elevated relative glucose concentration compared to controls (*p* < 0.05).

**Discussion:**

The *dcr* mice show evidence of significant metabolic dysregulation and add to the existing literature on the metabolic consequences of neurodegeneration. This work motivates future studies to understand the connection between mitochondrial dysfunction, metabolic alterations and neurodegeneration using the *dcr* mouse model.

## 1 Introduction

Metabolic processes in the brain are critical for brain function and health. Metabolomics studies, the measurement of low molecular weight biomolecules, investigate the underlying biochemical activity of cells, tissues or organisms and contribute to our understanding of system biology. In the human brain, metabolomics has revealed links between metabolic dysfunction and neurodegenerative diseases (e.g., Alzheimer’s disease, Parkinson’s disease, Huntington’s disease) and psychiatric disorders (e.g., bipolar disorder, major depressive disorder, schizophrenia) (Gonzalez-Riano et al., 2016). While it is challenging to study the molecular and cellular changes that occur in these disorders in humans, experimental animal models can provide insight into the pathophysiology.

The decrepit (*dcr*) mouse model of neurodegeneration resulted from a spontaneous mutation in a colony of C57BL6/J mice at Jackson Laboratories (Sproule et al., 2010). These mice display reproducible, adult-onset (70 days of age) degeneration of the brain that leads to premature death (by 150 days of age). With no evidence of amyloid *β* plaques or tau in the *dcr* brains, the model is distinct from Alzheimer’s disease and may represent a not yet identified human neurodegenerative disease. Using magnetic resonance imaging and immunohistochemistry, the brain lesion was shown to progress in a stereotypical neuroanatomical pattern via cell death along the metabolically active limbic system network (Cahill et al., 2020). At the end of the disease time course, the *dcr* mice show a greater than 30% decrease in the volume of the hippocampus compared to controls and demonstrate an associated memory impairment. Blood flow to the brain of *dcr* mice is abnormal and differs based on biological sex (Russell et al., 2025). Female *dcr* mice showed an increase in carotid artery blood flow with disease progression, while male *dcr* mice showed no change with age. These phenotypes result from an intronic mutation identified in a largely unstudied gene, mitochondrial ribosomal protein L3 (*Mrpl3*). A mutation in a gene related to energy metabolism and a reproducible pattern of neurodegeneration across linked brain regions makes the *dcr* mice a useful model to study metabolic pathways in neurodegeneration. We hypothesize that the consequences of the *Mrpl3* mutation will impact metabolites involved in ATP production, such as creatine, glucose and amino acids such as glutamine.

Metabolomics of unprocessed *ex vivo* brain tissue avoids solvent effects, allows for high sensitivity and resolution of the resulting data, and has been used previously to study neurodegeneration in mice (Kaebisch et al., 2017). In the present study, we used established ^1^H magic angle spinning nuclear magnetic resonance (MAS NMR) methods and *ex vivo* tissue samples to study the metabolome in the brains of female and male *dcr* and healthy control mice throughout adulthood. Characterizing the brain metabolic alterations in a novel mouse model of neurodegeneration will improve our understanding of the relationship between metabolism and neurodegeneration and may identify metabolic signatures of disease.

## 2 Materials and methods

### 2.1 Animal model

The *dcr* mouse model resulted from a spontaneous mutation in a colony of C57BL6/J mice (Sproule et al., 2010). The mutation is an insertion of at least 133 bp in a mitochondrial-associated gene (mitochondrial ribosomal protein L3, *Mrpl3*) (Cahill et al., 2020). The mice were rederived at The Centre for Phenogenomics (Toronto, Ontario) and bred in-house at the Health Sciences Centre Animal Facility at Memorial University of Newfoundland. Mice were co-housed in standard cages on a 12 h light:dark cycle and were given *ad libitum* access to water and food. For this study, 85 homozygous *dcr* mice (44 females and 41 males) and 79 controls (without the *dcr* mutation) (40 females and 39 males) were used. All animal experiments were approved by the Animal Care Committee at Memorial University of Newfoundland (Animal Use Protocol 21-01-LC) and conducted in accordance with guidelines from the Canadian Council on Animal Care.

### 2.2 Genotyping

Tail samples were collected for each mouse and DNA was extracted using an alkaline lysis method. Briefly, each sample was treated with 250 μL of 50 mM NaOH, incubated at 98 °C for 30 min and then vortexed vigorously. The samples were centrifuged at 15,000 rcf for 5 min at room temperature and 20 μL of the resulting supernatant was diluted with 80 μL of TE buffer (10 mM Tris–HCl, 1 mM disodium EDTA, pH 8.0). A total of 2 μL of the diluted DNA template was used for polymerase chain reaction (PCR) analysis with NEB Taq Polymerase (New England Biolabs, Whitby, ON, Canada). The PCR reaction mix contained 13.4 μL of nuclease-free water, 1.2 μL of MgCl_2_, 2 μL of buffer (10 mM Tris–HCl, 50 mM KCl, 1.5 mM MgCl_2_, pH 8.3), 0.4 μL of dNTPs, 0.4 μL of each primer, and 0.2 μL of Taq polymerase. The primers were Jax-F (TCT CGA GAG TCA GCT CAT AGA GAC A), Insertion-F (CAG GAA CAC CTC GAT GCT C), and Jax-R (CCC CGC AGA GAC TCA TTA CCT). The PCR cycling conditions were denaturation at 95 °C for 3 min, 35 cycles of 95° C for 30 s, 58 °C for 15 s, 72 °C for 1 min, with a final hold at 4 °C. The PCR products were analyzed on agarose gels with homozygous knockout mice expected to yield two bands at 214 bp and ~600 bp, heterozygous (Het) mice yielded three bands at 214 bp, 451 bp, and ~600 bp and control mice yielded a single band at 451 bp.

### 2.3 Experimental design

To assess brain metabolism throughout disease progression in the *dcr* mouse model of neurodegeneration, brain samples were collected in adulthood from 44 (prior to the onset of neurodegeneration) to 145 days of age (late stages of neurodegeneration and humane endpoint). The animals were selected to give uniform coverage across ages for both genotypes.

### 2.4 Magic angle spinning nuclear magnetic resonance

**Figure 1:**
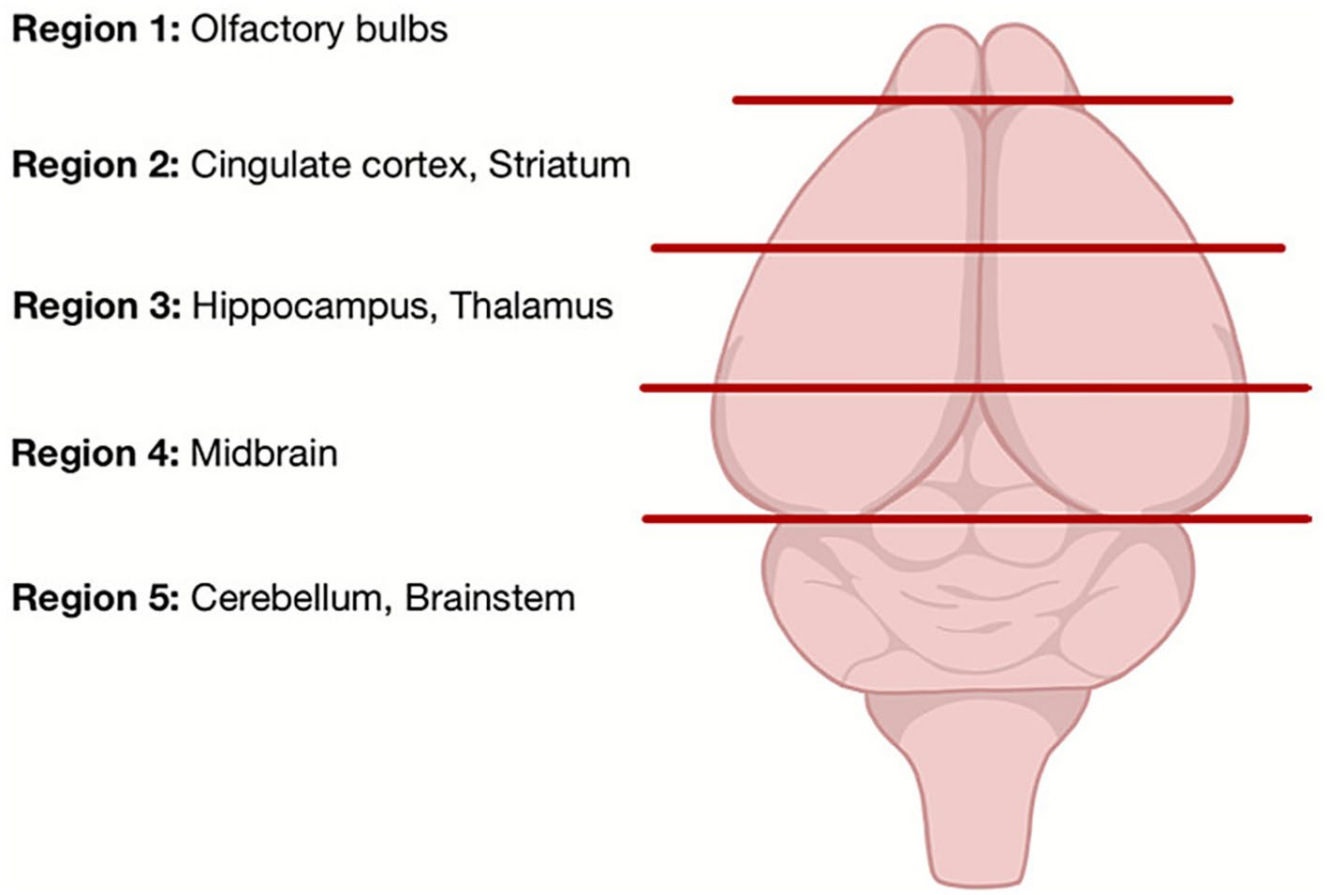
Representation of the five coronal brain regions dissected for ^1^H MAS NMR experiments.

The mice were weighed and then euthanized using decapitation under isoflurane anesthesia. The brain was removed from the skull and immediately frozen in liquid nitrogen to prevent metabolite degradation (Waters et al., 2000; Beckonert et al., 2010). The time from decapitation to freezing is less than 45 s. The samples were stored at −80 °C until preparation for NMR experiments. To minimize thawing, sample dissection was done keeping the tools and tissue cold with dry ice. The whole brain was cut down the mid-sagittal plane and then dissected into five anatomical regions using pre-defined landmarks (Figure 1). Each sample was packed into a 3.2 mm zirconium rotor (Cortecnet, Voisins-le-Bretonneux, France). ^1^H MAS NMR was performed using a H-C-P 3.2 mm MAS triple-tuned solid-state Bruker NMR probe (spinning rate = 4 kHz) and a Bruker Avance II 600 MHz spectrometer (^1^H Larmor frequency = 600.29 MHz). A water presaturation pulse was used for suppression of the water signal and experiments were acquired with a bearing gas temperature of 37 °C, a 90° pulse (length = 3 μs), a recycle delay of 5 s, and 32 scans (Schneider et al., 2022). Eighty-one (44 *dcr* and 37 control) spectra (10% of total acquisitions) had to be excluded because of incorrect tissue dissection, low signal-to-noise ratio or instrumentation failure during the acquisition.

### 2.5 Data processing

All NMR spectra were processed using MestReNova (version 15.1.0, Mestrelab Research, S.L., Norwich, CT). Each spectra were referenced to the water resonance (before suppression) and automated phase and baseline correction using the splines function were applied, followed by manual phase correction, if needed. Using the automatic peak picking function and spectral deconvolution, the resonances for each metabolite were integrated. This approach for determining peak intensities detects only the narrow resonances from low molecular weight metabolites and does not include broad resonances from lipids. The integrated regions were normalized to the integral region between 0.0–6.0 ppm (excluding the water peak). Normalization (to give relative metabolite concentrations) accounts for differences in the weight of the brain tissue samples and the tissue water content. Literature values (Ghosh et al., 2012; Govindaraju et al., 2000; Graham et al., 2016; Mercer et al., 2023; Schenetti et al., 2006) and correlations from a 2D ^1^H-^1^H Correlation spectrum were used to assign the ^1^H resonances.

### 2.6 Statistical analysis

Statistical tests were performed using the R software package (https://www.r-project.org) and the R packages ggplot2 (Wickham, 2016), nlme (Pinheiro et al., 2025), lme4 (Bates et al., 2015) and multcomp (Hothorn et al., 2008). The body weight growth curves were assessed with a best fit quadratic model with main effects of age, genotype, and sex and including interaction terms. The metabolomics data was analyzed using a best fit linear or quadratic model (a quadratic model was statistically justified for 2 of 14 metabolites) and a linear mixed-effects model with main effects of age, genotype, sex, and brain region and including interaction terms and mouse ID as the random effect. To investigate sex-specific differences, the data was disaggregated into females and males and analyzed using a linear mixed-effects model with main effects of age, genotype, and brain region and including interactions terms and mouse ID as the random effect. Pooling the female and male data together may prevent detection of meaningful sex differences and presentation of the raw data can facilitate a future meta-analysis by sex (Tannenbaum et al., 2019). Multiple comparisons were tested using a False Discovery Rate of 10% and statistical significance was defined as *p* < 0.05. A False Discovery Rate of 10% is considered appropriate for metabolomics studies when the aim is to postulate novel hypotheses and 5% is used for more rigorous multiple comparison control (Alka et al., 2022; Shanthamoorthy, 2022). If the linear mixed-effects model was significant, additional *post hoc* tests were performed. Total variation in relative metabolite concentration was modeled as a sum of inter- and intra-variation, with the parameters estimated using the reduced maximum likelihood algorithm.

To explore differences in entire metabolic profiles, multivariate analysis was conducted using MetaboAnalyst (version 6.0) (Pang et al., 2024) and unsupervised Principal Component Analysis (PCA). In a 2D scores plot, the separation between genotypes was analyzed using a one-way analysis of variance. To determine the relevance of metabolic abnormalities on biological processes, a metabolic pathway analysis was conducted using MetaboAnalyst. The data was analyzed using the compound list option (KEGG ID), the *Mus musculus* pathway library and the list of metabolites that were significantly different between *dcr* and control mice. Statistical significance was defined as *p* < 0.05. For each pathway, the impact score and match status (hit/total) is reported, with hit representing the number of compounds in the total pathway that matched the list of metabolites that were altered in the *dcr* mice. Only pathways with an impact score > 0.0 are considered of topological importance.

## 3 Results

The *dcr* mice disease onset occurs at 70 days of age, with the first evidence of neurodegeneration in the entorhinal and piriform cortex on magnetic resonance images (Cahill et al., 2020) and evidence of neuronal death in the cortex at 84 days (Sproule et al., 2010). After 70 days of age the mice cease to gain weight compared to controls (age-by-genotype interaction, *p* = 0.0005) (Supplementary Figure 1). The female *dcr* mice slowly continue to gain weight until the end of their lifespan, while the male *dcr* mice show no increase in weight with age after disease onset.

**Figure 2:**
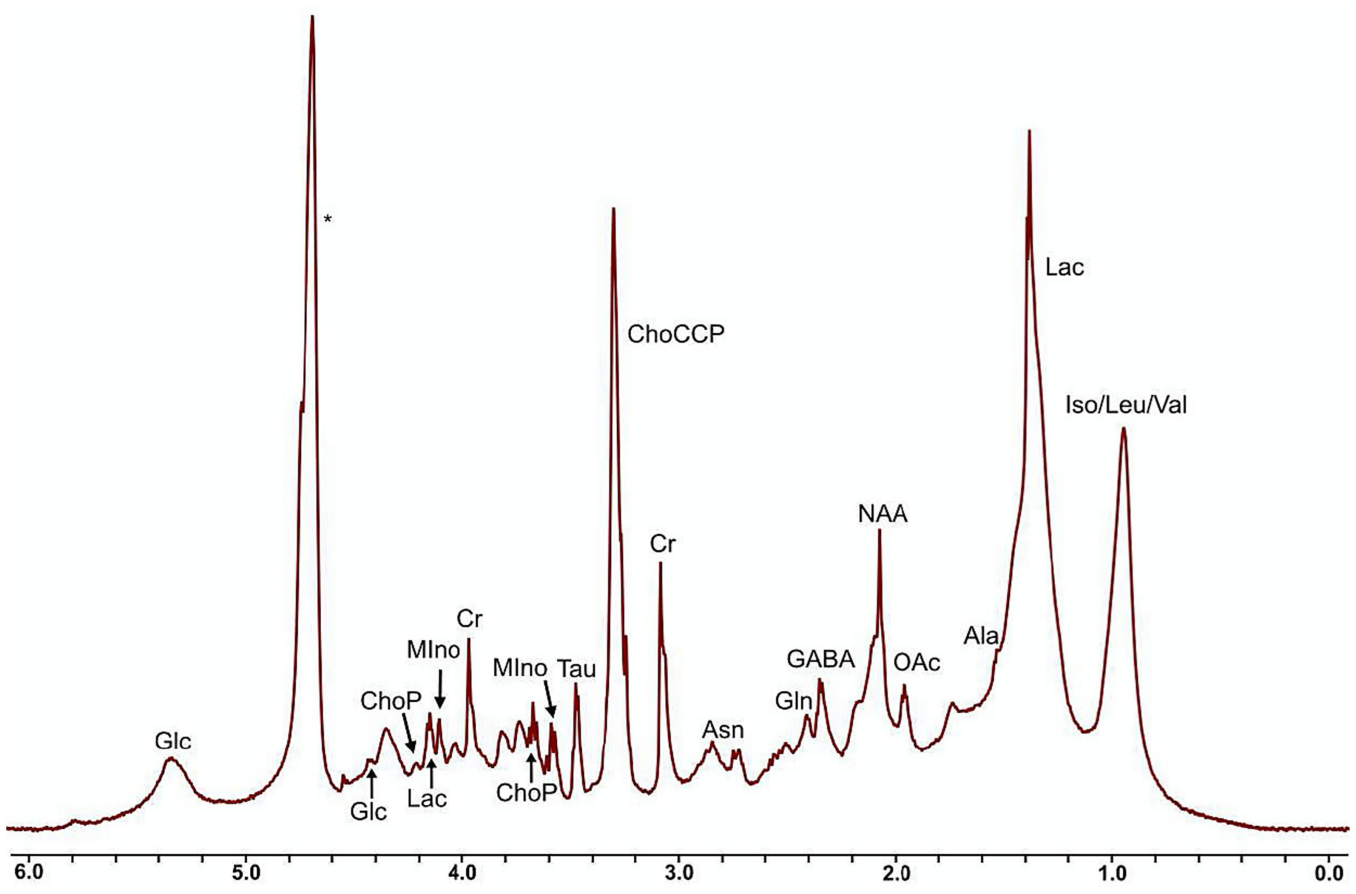
^1^H NMR spectra of the murine adult brain tissue sample (water presaturation pulse, MAS = 4 kHz, 37 °C, 32 scans). Ala, alanine; Asn, asparagine; ChoCCP, choline-containing compounds; ChoP, phosphocholine; Cr, creatine; GABA, gamma-aminobutyric acid; Gln, glutamine; Glc, glucose; Iso, isoleucine; Lac, lactate; Leu, leucine; MIno, myo-inositol; NAA, N-acetylaspartate; Oac, acetate; Tau, taurine; Val, valine; *water.

**Figure 3:**
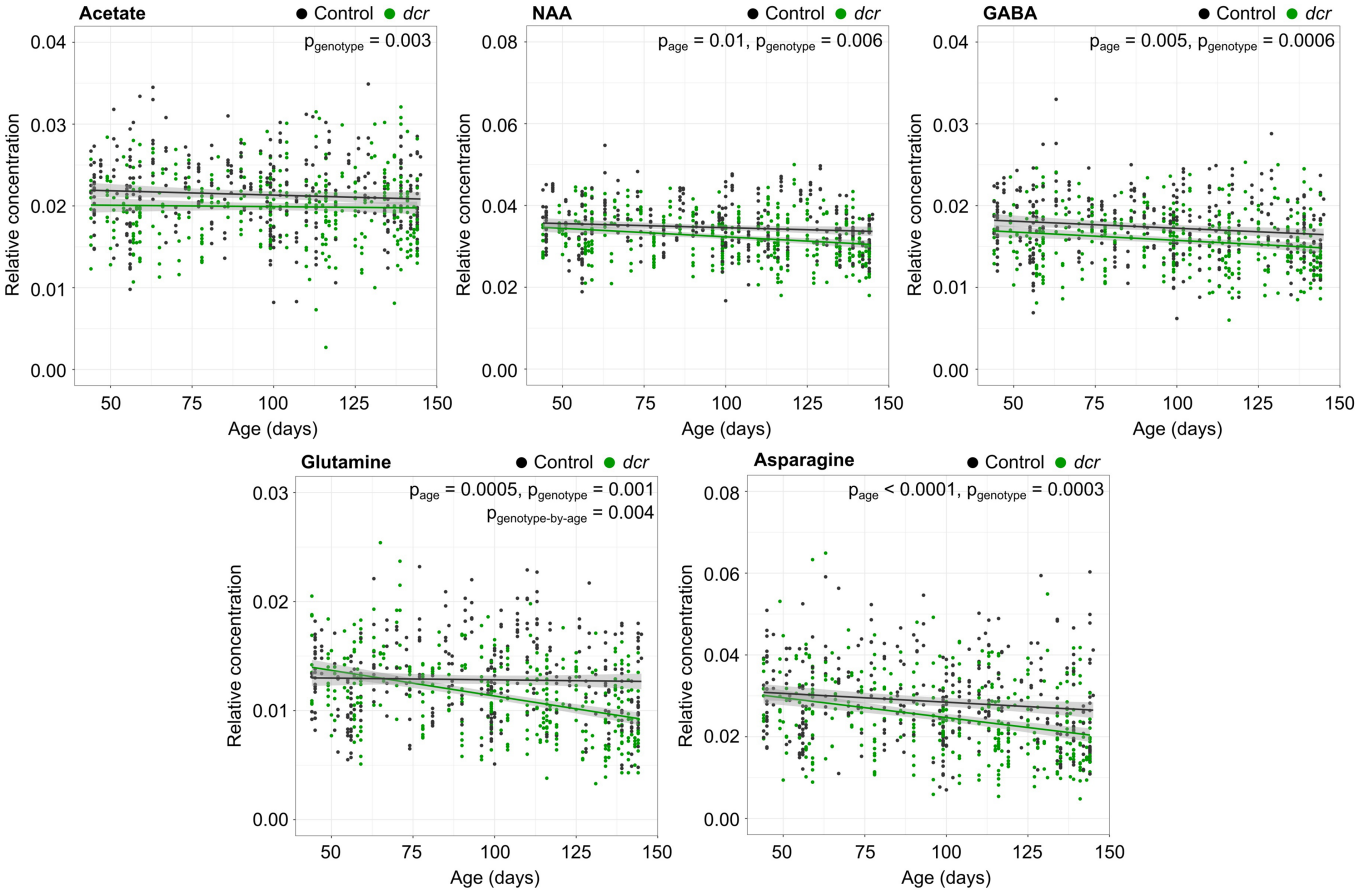
Differences in relative metabolite concentration between *dcr* (green) and control (black) mice with age. The relative concentrations were fit using a linear model. Main effects of age and genotype are noted as *p*_age_, *p*_genotype,_ and genotype-by-age interactions are noted as *p*_genotype-by-age_. *n* = 39–44 mice/genotype/sex. The shaded gray area represents the 95% confidence interval.

**Figure 4:**
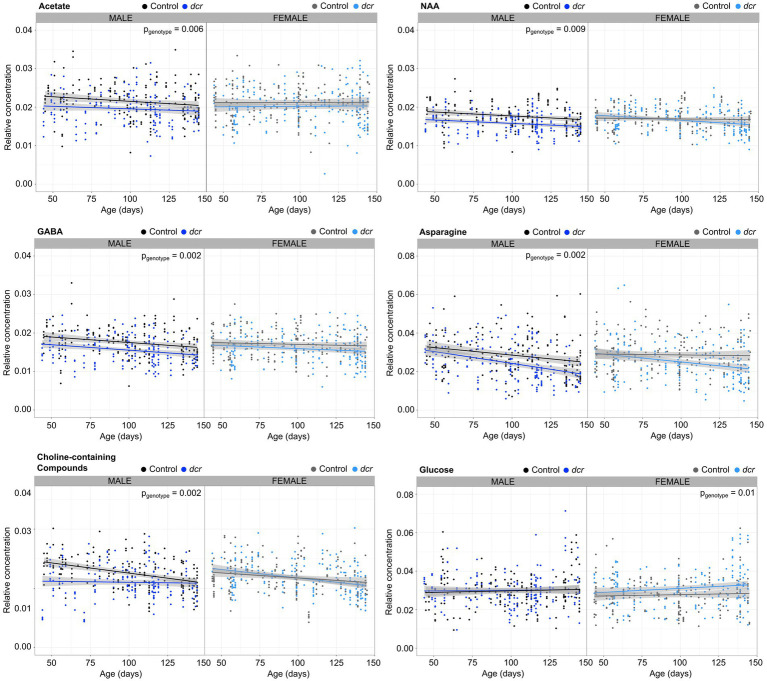
The difference in the relative metabolite concentrations between control (male: black, female: gray) and DCR (male: dark blue, female: light blue) mice depended on sex. Main effect of genotype is noted as p_genotype_. *n* = 39–44 mice/genotype/sex. The shaded gray area represents the 95% confidence interval.

**Figure 5:**
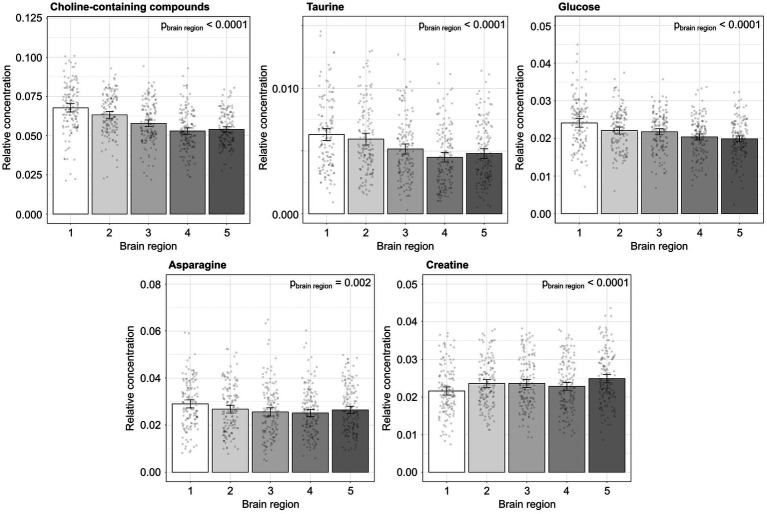
The relative concentration of metabolites that showed a significant main effect of brain region [1: rostral (olfactory bulbs) to 5: caudal (cerebellum/brainstem)]. The main effect of brain region is noted as *p*_brain region_. *n* = 39–44 mice/genotype/sex. The data is presented as means and 95% confidence intervals.

In the ^1^H NMR spectra of the murine adult brain (Figure 2), the metabolites isoleucine, leucine and valine could not be clearly resolved and were grouped together. Thirteen additional metabolites or classes of metabolites (choline-containing compounds) were assigned and quantified in all of the brain samples. Relative metabolite concentrations were compared with age, genotype, sex and brain region. Table 1 summarizes the main effects of these comparisons and the most significant results are described below and presented in Figures 3–5. Of the 14 metabolites measured, there was a significant main effect of age for 8 of the metabolites. The relative concentrations of N-acetylaspartate (NAA), gamma-aminobutyric acid (GABA), glutamine, asparagine, creatine, choline-containing compounds, taurine and phosphocholine decreased with age. Significant region-by-age interactions were found for glutamine and asparagine and *post hoc* tests showed the relative concentrations of glutamine and asparagine only varied with age in 4 of the 5 brain regions (not in the midbrain). For 5 of the 14 metabolites there was a significant main effect of genotype, with a decrease in the relative concentration in acetate, NAA, GABA, glutamine, and asparagine in *dcr* mice compared to controls. A significant genotype-by-age interaction demonstrated that the relative concentration of glutamine decreased significantly with age in the *dcr* mice while there was no change with age in the control mice. To specifically investigate differences between females and males, the data was disaggregated by sex and analyzed separately. This analysis showed that in male *dcr* mice, acetate (*p* = 0.006), NAA (*p* = 0.0009), GABA (*p* = 0.002), asparagine (p = 0.002), and choline-containing compounds (*p* = 0.002) were decreased compared to controls. In females, *dcr* mice had a significant increase in the relative concentration of glucose (*p* = 0.01) compared to controls.

**Table 1:** Main effects for variables modeled as a four-way interaction between age, genotype, sex and brain region.

Metabolite	Age	Genotype	Brain region	Interaction
Isoleucine/leucine/valine	NS	NS	NS	NS
Lactate	NS	NS	*F* = 3.9, *p* = 0.004**	NS
Alanine	NS	NS	*F* = 4.7, *p* = 0.001**	NS
Acetate	NS	*F* = 8.8, *p* = 0.003** Decreased in *dcr.*	NS	NS
N-acetylaspartate	*F* = 6.8, p = 0.01* Decreased.	*F* = 7.8, p = 0.006* Decreased in *dcr.*	NS	NS
Gamma-aminobutyric acid	*F* = 8.1, *p* = 0.005* Decreased.	*F* = 12.2, *p* = 0.0006** Decreased in *dcr.*	NS	NS
Glutamine	*F* = 12.6, p = 0.0005** Decreased.	*F* = 10.8, p = 0.001** Decreased in *dcr.*	NS	Genotype-by-age: *F* = 8.5, p = 0.004** Decreased in *dcr*. Region-by-age: *F* = 3.6, p = 0.006*
Asparagine	*F* = 19.4, p < 0.0001** Decreased.	*F* = 14.0, *p* = 0.0003** Decreased in *dcr.*	*F* = 4.5, p = 0.002** Increased in region 1 vs. 3, 4.	Region-by-age: *F* = 3.5, *p* = 0.008*
Creatine	*F* = 15.1, *p* = 0.0002** Decreased.	NS	*F* = 13.8, p < 0.0001** Deceased in region 1 vs. 2, 3, 5.	NS
Choline-containing compounds	*F* = 16.8, *p* = 0.0001** Decreased.	NS	*F* = 110.7, p < 0.0001** Decreased from rostral to caudal.	NS
Taurine	*F* = 10.2, p = 0.002** Decreased.	NS	*F* = 27.4, p < 0.0001** Decreased from rostral to caudal.	NS
Myo-inositol	NS	NS	*F* = 3.4, p = 0.01*	NS
Phosphocholine	*F* = 14.8, p < 0.0001** Decreased.	NS	NS	NS
Glucose	NS	NS	*F* = 10.3, p < 0.0001** Increased in region 1.	NS

The uncorrected *p*-values are marked with an asterisk if significant at a false discovery rate of 10% and two asterisk if significant at a false discovery rate of 5%.

NS, not significant.

There was a main effect of brain region for the relative concentration of 8 metabolites including lactate, alanine, asparagine, creatine, choline-containing compounds, taurine, myo-inositol and glucose. *Post hoc* analysis showed the relative concentrations of choline-containing compounds and taurine decreased significantly from rostral to caudal in the brain, while asparagine and glucose showed a significant increase in only the olfactory bulb and creatine showed a significant decrease in the olfactory bulb compared to other brain regions. For the metabolite concentrations, the intra-animal standard deviation (which incorporates true variation between regions of the same brain and experimental error) was similar to the inter-animal standard deviation. This means the brains from different mice have approximately double the variation and the variability caused by anatomical brain regions and experimental error from MAS NMR is small compared to the biological variability of the *dcr* mouse model.

Multivariate analysis using unsupervised PCA showed no statistically significant separation of the *dcr* and control brains for the first and second principal components (*p* > 0.05). The first two principal components represented 79.8% of the total variation. The metabolic pathway analysis showed the *dcr* mice have a significant perturbation in one biochemical pathway in the brain: alanine, aspartate and glutamate metabolism (*p* < 0.0001, impact score = 0.29, match status = 4/28).

## 4 Discussion

Using NMR metabolomics, the relative brain metabolite concentrations of acetate, NAA, GABA, glutamine, and asparagine were found to be decreased in the *dcr* mice compared to controls. There was no difference between the *dcr* and control mice in the PCA of the entire metabolite profile, indicating that the metabolic disruption is specific to these metabolites alone. All of the metabolic changes occur after the onset of disease (70 days of age), providing information on the metabolic pathways that are associated with neurodegeneration. NAA, a marker of neuronal integrity, is often found to be reduced in human neurodegenerative diseases such as Pick disease (Cheng et al., 1997), Alzheimer’s disease (Chen et al., 2000; Huang et al., 2001) and amyotrophic lateral sclerosis (Hanstock et al., 2020). Similar to the decrease in NAA observed in this study, transgenic mouse models of Alzheimer’s disease and Huntington’s disease have also found decreases in NAA concentrations (Jenkins et al., 2000; Salek et al., 2010). Acetate concentration has been shown to correlate with NAA concentration in neurodegenerative brain samples (Li et al., 2013). Two neurotransmitters, GABA (inhibitory) and glutamine (excitatory) were significantly decreased in the *dcr* mice compared to controls. Abnormal neurotransmitter levels could indicate neurotoxic cell damage. GABA and glutamine are altered in neurodegenerative diseases and associated with cognitive and physical decline (Błaszczyk, 2016; Bornstein et al., 2023; Cawley et al., 2015) such as the memory impairment and abnormal behavior found in the *dcr* mice (Cahill et al., 2020; Sproule et al., 2010). Asparagine is an essential amino acid important for brain development and function and decreased levels have been previously associated with increased cell death (Ruzzo et al., 2013). This is consistent with the known brain phenotype in the *dcr* mice, with cell death reported in the cortex (cingulate, piriform, entorhinal cortices), the amygdala, and the hippocampus (Sproule et al., 2010; Cahill et al., 2020).

Differences in energy demands and tissue structures means that the concentration of metabolites are expected to vary in different anatomical brain regions (Cichocka et al., 2016; Minati et al., 2010). While there was a main effect of brain region in 8 of the 14 metabolites, there were no significant genotype-by-region interactions, demonstrating that one specific brain region is not more susceptible to metabolic damage in the *dcr* mice. Moreover, compared to the differences within mice, the variability between mice and between genotypes was larger. Choline and taurine concentrations showed a significant decrease from rostral to caudal regions of the brain. While we did not anticipate this result, the distribution may be related to the functional specialization and the neuronal complexity of the brain regions. For example, rostral brain areas may require higher levels of choline for acetylcholine synthesis due to their involvement in sensory processing and higher order functions.

While recent evidence suggests abnormal metabolite levels may precede the onset of symptoms and neurodegeneration (Muddapu et al., 2020), there were no significant differences in metabolite concentrations between *dcr* and control mice prior to the onset of disease (70 days of age). This suggests the abnormal metabolism is not the causative mechanism but instead the consequence of the neurodegeneration in the *dcr* mice. However, the study design was cross-sectional and therefore the analysis of the temporal dynamics within individual mice is indirect. We cannot conclusively rule out metabolic involvement in disease initiation in the *dcr* mice. It is likely that there is a bidirectional relationship between the genetic dysfunction (mitochondrial-associated neurodegeneration) and the metabolite alterations.

The present study has several additional limitations. While the time from sample preparation of the tissue to data acquisition is consistent from sample-to-sample and short (<10 min), *ex vivo* experiments are susceptible to biochemical changes and tissue degradation that are not a concern with *in vivo* studies (Beckonert et al., 2010). The cell death that occurs in the *dcr* mice could confound the relative metabolite changes and could be addressed in future studies with the use of internal standards for absolute quantification. The brain was subdivided into five anatomical regions and while these regions were reproducible between samples, the regions are crude and lack structural specificity. This anatomical coarse graining may have masked region-specific effects. Future studies will use cryo-dissection techniques and investigate the metabolic changes in specific brain structures such as the hippocampus, entorhinal cortex and amygdala. The present study was limited to the detection of 14 metabolites because of the use of a standard solid-state MAS NMR probe. The spectral line broadening could be reduced using advanced pulse sequences [e.g., Carr-Purcell-Meiboom-Gill experiments (Lucas et al., 2005)] and using a high-resolution MAS NMR probe designed specifically for biological tissue samples (with a deuterium lock channel and a gradient coil along the magic angle). Finally, 10% of the NMR spectra had to be excluded from this study. While the balance was similar between the *dcr* and control mice, data exclusion may introduce bias.

## 5 Conclusion

NMR metabolomics is a promising method to study brain health and reveal the underlying molecular biochemical processes altered during neurodegeneration. The *dcr* mouse model undergoes tissue degeneration and significant alterations in brain metabolism. Magnetic resonance imaging is routinely used in the diagnosis of neurodegenerative diseases. The present study identified several brain metabolic changes associated with neurodegeneration that could be explored in future studies to improve prediction and diagnosis. While the *Mrpl3* gene that carries the mutation in the *dcr* mice has unknown function, future studies should determine mitochondrial function and use electrophysiology to investigate synaptic function in the *dcr* mice.

## Data Availability

The raw data supporting the conclusions of this article will be made available by the authors, without undue reservation.

## Supplementary material

Supplementary Figure 1 Change in body weight for control (female: black, male: gray) and dcr (female: green, male: blue) mice from 44 to 145 days of age. Main effects of age, genotype and sex are noted as *p*_age_, *p*_genotype_ and *p*_sex_ and the genotype-by-age, genotype-by-sex and genotype-by-age-by-sex internactions as *p*_genotype_-by-age, *p*_genotype_-by-sex and *p*_genotype_-by-age-sex. *n*=39-44 mice/genotype/sex.
