# Supplementary figures and images for: NMR metabolomics reveals metabolic alterations in a novel mouse model of neurodegeneration
Source: Front Neurosci. 2026 Feb 12;20:1776973. doi: 10.3389/fnins.2026.1776973 (PMC12936862; doi:10.3389/fnins.2026.1776973)

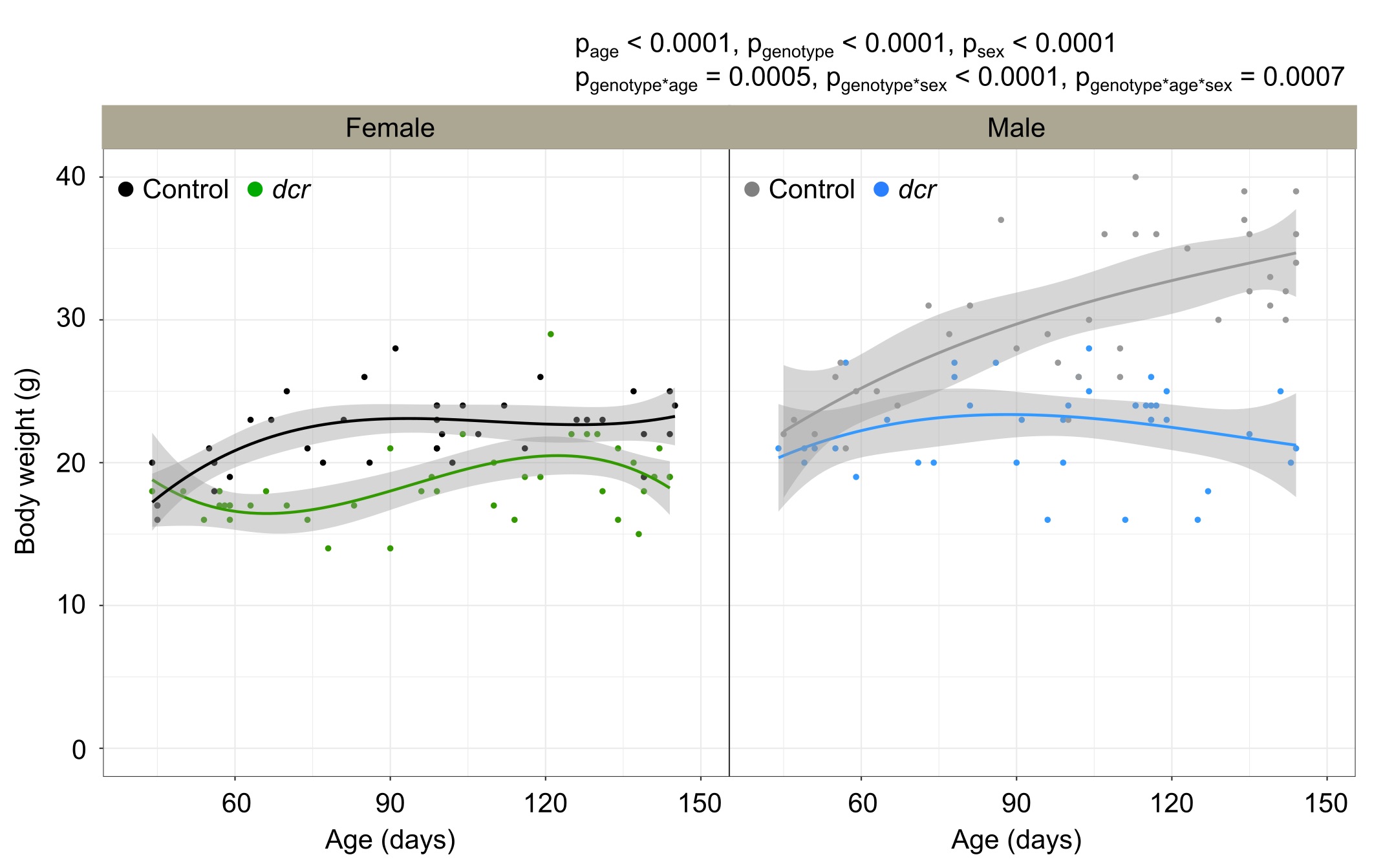

Supplement: Supplementary Figure 1 — Change in body weight for control (female: black, male: gray) and dcr (female: green, male: blue) mice from 44 to 145 days of age. Main effects of age, genotype and sex are noted as page, pgenotype and psex and the genotype-by-age, genotype-by-sex and genotype-by-age-by-sex internactions as pgenotype-by-age, pgenotype-by-sex and pgenotype-by-age-sex. n=39-44 mice/genotype/sex. [file Image_1.jpg]
